# Overexpression of VEGFα as a biomarker of endothelial dysfunction in aortic tissue of α-GAL-Tg/KO mice and its upregulation in the serum of patients with Fabry’s disease

**DOI:** 10.3389/fcvm.2024.1355033

**Published:** 2024-02-05

**Authors:** N. Lund, H. Wieboldt, L. Fischer, N. Muschol, F. Braun, T. Huber, D. Sorriento, G. Iaccarino, K. Müllerleile, E. Tahir, G. Adam, P. Kirchhof, L. Fabritz, M. Patten

**Affiliations:** ^1^ Department of Cardiology, University Heart & Vascular Center Hamburg, University Medical Center Hamburg-Eppendorf, Hamburg, Germany; ^2^ Department of Intensive Care Medicine, University Hospital Hamburg-Eppendorf, Hamburg, Germany; ^3^ Department of Pediatrics, University Medical Center Hamburg-Eppendorf, Hamburg, Germany; ^4^ III. Department of Medicine, University Medical Center Hamburg-Eppendorf, Hamburg, Germany; ^5^ Hamburg Center for Kidney Health, University Medical Center Hamburg-Eppendorf, Hamburg, Germany; ^6^ Department of Advanced Biomedical Sciences, Interdepartmental Center of Research on Hypertension and Related Conditions of the Federico II University, Naples, Italy; ^7^ Department of Clinical Medicine and Surgery, Interdepartmental Center of Research on Hypertension and Related Conditions of the Federico II University, Naples, Italy; ^8^ Department of Diagnostic and Interventional Radiology and Nuclear Medicine, University Medical Center Hamburg-Eppendorf, Hamburg, Germany

**Keywords:** Fabry’s disease, vasculopathy, endothelial dysfunction, angiogenic markers, VEGF, angiostatin, aortic vessels, transgenic knockout model

## Abstract

**Introduction:**

Fabry's disease is an X-linked lysosomal storage disorder caused by reduced activity of α-galactosidase A (GAL), leading to premature death on account of renal, cardiac, and vascular organ failure. Accumulation of the GAL substrate globotriaosylceramide (Gb3) in endothelial and smooth muscle cells is associated with early vascular cell damage, suggesting endothelial dysfunction as a driver of cardiorenal organ failure. Here, we studied the vascular expression of the key angiogenic factors, VEGFα and its antagonist angiostatin, in Fabry α-GAL-Tg/KO mice and determined circulating VEGFα and angiostatin serum levels in patients with Fabry’s disease and healthy controls.

**Methods:**

Cryopreserved aortic vessels from six α-GAL-Tg/KO and six wild-type (WT) mice were obtained and VEGFα and angiostatin levels were determined by performing Western blot analysis. VEGFα expression was visualized by an immunohistochemical staining of paraffin aortic rings. In addition, VEGFα and angiostatin serum levels were measured by using an enzyme-linked immunosorbent assay in 48 patients with genetically verified Fabry's disease (50% male) and 22 healthy controls and correlated with disease severity markers such as lyso-Gb3, albuminuria, NTproBNP, high-sensitive troponin T (hsTNT), and myocardial wall thickness.

**Results:**

It was found that there was a significant increase in VEGFα protein expression (1.66 ± 0.35 vs. 0.62 ± 0.16, *p* = 0.0009) and a decrease in angiostatin expression (0.024 ± 0.007 vs. 0.053 ± 0.02, *p* = 0.038) in aortic lysates from α-GAL-Tg/KO compared with that from WT mice. Immunohistochemical staining revealed an adventitial VEGFα signal in α-GAL-Tg/KO mice, whereas no VEGFα signal could be detected in WT mice aortas. No differences in aortic angiostatin expression between α-GAL-Tg/KO- and WT mice could be visualized. The serum levels of VEGFα were significantly upregulated in patients with Fabry’s disease compared with that in healthy controls (708.5 ± 426.3 vs. 458.5 ± 181.5 pg/ml, *p* = 0.048) and positively associated with albuminuria (*r* = 0.82, *p* < 0.0001) and elevated NTproBNP (*r* = 0.87, *p* < 0.0001) and hsTNT values (*r* = 0.41, *p* = 0.048) in male patients with Fabry’s disease. For angiostatin, no significant difference was found between patients with Fabry’s disease and healthy controls (747.6 ± 390.3 vs. 858.8 ± 599.3 pg/ml).

**Discussion:**

In conclusion, an overexpression of VEGFα and downregulation of its counter player angiostatin in aortic tissue of α-GAL-Tg/KO mice support the hypothesis of an underlying vasculopathy in Fabry's disease. Elevated VEGFα serum levels were also observed in patients with Fabry’s disease and were positively associated with elevated markers of organ manifestation in males. These findings suggest that angiogenetic markers, such as VEGFα, may be potentially useful biomarkers for the detection of endothelial dysfunction in classical Fabry's disease.

## Introduction

Fabry's disease is an X-linked lysosomal storage disorder caused by reduced or absent α-galactosidase A (GAL) activity resulting from mutations in the *GLA* gene (1). Subsequently, globotriaosylceramide (Gb3) and its deacylated form, globotriaosylsphingosine (lyso-Gb3), accumulate in different cells and tissues throughout the body causing various clinical manifestations.

Endothelial and smooth muscle cells are the preferential target of Gb3 storage (2). Gb3 induces a variety of angiogenesis factors such as VEGF, VEGFR2, TGFß, and FGF-2 in these cells (3). Endothelial dysfunction was described in experimental models of Fabry's disease (4, 5) and may also play a pivotal role in affected patients with Fabry’s disease (6). Endothelial dysfunction (6) and vascular damage reflected by increased intima-media thickness (IMT) and decreased brachial flow–mediated dilation (7) also appear to contribute to organ dysfunction in patients with Fabry’s disease, including chronic kidney disease, cardiomyopathy, and stroke (8).

VEGFα is a specific endothelial cell mitogen acting as a potent pro-angiogenic factor (9). Its antagonist, angiostatin, attenuates VEGF expression, resulting in the inhibition of extracellular matrix formation and migration (10, 11). VEGFα is activated in a variety of kidney, heart, cerebral, and cutaneous diseases associated with angiogenesis (12–15). In this context, prior work from our group suggests altered markers for vascular dysfunction and shear stress in the blood of patients with Fabry’s disease (16).

Here, we studied the vascular expression pattern and localization of VEGFα and angiostatin in aortic vessels of Fabry R301Q transgenic/KO (α-GAL-Tg/KO) mice. In addition, VEGFα and angiostatin concentrations were determined in the serum of patients with Fabry’s disease and healthy controls and were found to be associated with biomarkers of disease severity.

## Materials and methods

### Mouse model

Experiments were carried out in C57BL/6 (wild-type, WT) and hR301Q α-GAL-Tg/KO mice. Specifically, these mice are homozygous for endogenous GLA knockout and express the human transgene GLA carrying the R301Q mutation under the transcriptional control of the human GLA promoter. These mice were kindly provided by AMICUS Therapeutics and used as a model of Fabry’s disease (17–19). The mice were housed in the animal facility of the Department of Translational Medical Sciences of Federico II University of Naples (Italy) in a 12-h light–dark cycle and provided access to a commercial mouse diet and water *ad libitum*. Animal husbandry and other experiments were conducted under Institutional Animal Care and the use of committee-approved protocols at Federico II University (Prot. n. 971/2016-PR). Aortas were collected from 9-month-old mice. Mice were euthanized, and aortas were quickly removed, rinsed in cold phosphate-buffered saline (PBS), and processed for successive analyses: one part was immediately frozen in liquid nitrogen for a biochemistry analysis, and another part was fixed in 4% paraformaldehyde for histological analysis.

### Tissue preparation (aortic vessels)

Cryopreserved aortic vessels from α-GAL-Tg/KO mice and WT mice were pulverized and homogenated on ice in a RIPA buffer supplemented with protease inhibitors by repeated sonication and incubation for 1 h. The homogenates were centrifuged for 30 min at 13,000 rpm at 4°C and protein content in the supernatants was measured using a BCA assay (Thermo Fisher).

### Western blotting

The total protein extracts (20 µg) were separated on TGX 4%–20% precast gradient gels (Bio-Rad, Germany) and transferred onto nitrocellulose membranes. The membranes were blocked in 5% milk (for anti-angiostatin antibody) or 3% milk (for anti-VEGFα and anti-α-actinin antibodies). The membranes were then incubated with the following primary antibodies:

Polyclonal rabbit-a-angiostatin (Abcam, ab2904) 2 µg/ml in 5% milk, overnight at 4°C; monoclonal mouse-a-VEGFα (Abcam, ab1316), 5 µg/ml in tris-buffered saline (TBS)/T, 1 h at room temperature; rabbit-a-α-actinin (Cell Signaling, 3134), 1:1,000 in 5% milk, overnight at 4°C. After washing with TBS/T, the membranes were incubated with horseradish peroxidase (HRP)-conjugated secondary antibodies (sheep anti-mouse, Jackson ImmunoResearch 515-035-003, 1:20,000 in 3% milk; goat anti-rabbit, Jackson ImmunoResearch JIM-111-035-003, 1:10,000 in 5% milk), washed again, and specific signals were detected using the ECL or Femto system (Thermo Fisher) according to the manufacturer's protocol. A densitometric analysis of the protein bands was done using ImageLab (Bio-Rad).

### Immunohistochemistry

Paraffin sections (3 µm) of aortic vessels from WT and α-GAL-Tg/KO mice were deparaffinized and incubated in a 10 mM citrate buffer at 95°C for 40 min for antigen retrieval. Afterward, the sections were incubated for 10 min in 0.5% Triton/TBS for permeabilization, washed in TBS, and quenched in a 0.25% Sudan black solution for 30 min on a shaker in the dark. After repeated washing, the sections were blocked in 3% bovine serum albumin (BSA) in TBS for 1 h at room temperature. The sections were then incubated with primary antibodies [anti-angiostatin (Abcam ab2904), 1:20 in 3% BSA or anti-VEGFα (Abcam, ab1316), 1:400 in 3% BSA] overnight at 4°C, washed in TBS, and incubated with secondary antibodies [donkey anti-mouse Alexa Fluor 488 (A21202 life tech) or donkey anti-rabbit Alexa Fluor 488 (21206 life tech), 1:500 in 3% BSA] for 2 h at room temperature. After repeated washing in TBS, the slides were mounted with DAPI Fluoromount G (Biozol) and fluorescence signals were descriptively analyzed using a Zeiss Axiovert M200 with ApoTome.

### Study population

In this retrospective cross-sectional study, a total of 48 patients with genetically verified Fabry's disease was included and compared with 22 healthy control patients. Participants were enrolled during routine visits at the outpatient clinic of the University Heart and Vascular Center Hamburg between January 2014 and December 2020. A medical history of coronary artery disease (CAD), diabetes mellitus, arterial hypertension, and stroke was confirmed by self-report or the use of corresponding medication. A diagnosis of atrial fibrillation (AF) was established by a positive history and electrocardiogram (ECG) documentation within the last 5 years prior to examination. Cardiac symptoms were classified according to the New York Heart Association (NYHA) classification. All participants received a 12-lead surface ECG, a transthoracic echocardiography, and a routine blood test including a measurement of hsTNT (high-sensitive troponin T) and NTproBNP (brain natriuretic peptide), creatinine, GFR, and albumin in urine. The serum levels of NTproBNP were assessed by using the Atellica® IM NT-proBNP assay (Siemens Healthcare, Erlangen, Germany). For the measurement of serum hsTNT levels, the Elecsys Troponin T hs STAT assay (Roche Diagnostics, Rotkreuz, Schweiz) was used. In 40 patients with Fabry’s disease and in all healthy controls, cardiac magnetic resonance (CMR) imaging was performed to determine the extent of fibrosis as a marker of Fabry-associated cardiomyopathy. The study was conducted in compliance with the principles outlined in the Declaration of Helsinki and was approved by the local ethics committee (Ethikkommission der Ärztekammer Hamburg, Nr.: PV4056). All study participants gave their written informed consent.

### Cardiac imaging

All study subjects underwent a comprehensive transthoracic echocardiographic examination (Philips iE33 system, Philips Healthcare, Best, Netherlands) including M-mode, two-dimensional, pulsed, and continuous-wave Doppler and tissue Doppler imaging. Structural and functional imaging parameters were measured according to the current recommendations of the American Society of Echocardiography and diastolic dysfunction was classified according to current guidelines (20). Images were analyzed using the commercially available software Syngo Dynamics (Siemens Healthcare, Erlangen, Germany).

CMR was performed on a 1.5-T scanner (Achieva, Philips Healthcare, Best, Netherlands). The imaging protocol included cine imaging and late gadolinium enhancement (LGE) imaging. LGE images were acquired using a standard phase-sensitive inversion recovery (PSIR) sequence at least 10 min after a bolus injection of gadoterate meglumine (Dotarem, Guerbet, Sulzbach, Germany) in three long-axis orientations (2CH, 3CH, and 4CH views) and a stack of short-axis slices. Typical imaging parameters were as follows: voxel size 0.98 mm^3^ × 0.98 mm^3^ × 8 mm^3^, echo time = 2.39 ms, time to repetition = 4.97 ms, and flip angle = 15°. The presence of LGE was assessed by using the commercially available software cvi42 (Circle Cardiovascular Imaging Inc., Calgary, Alberta, Canada).

### Quantification of VEGFα and angiostatin in patients’ serum

Protein serum levels in samples from patients with Fabry’s disease and healthy controls were quantified using enzyme-linked immunosorbent assay (ELISA) kits. For measuring and analyzing VEGFα serum levels, the ELISA kit BMS277-2TEN (Invitrogen) was used, and for determining angiostatin serum levels, the ELISA kit #ELH-Angiostatin (RayBio) was used, respectively, in accordance with the manufacturer's instructions.

### Statistical analysis

A statistical analysis was performed using IBM SPSS Statistics (Version 28.0, Statistical Package for the Social Sciences, International Business Machines, Inc., Armonk, NY, USA). Continuous data are given as mean and standard deviation (SD). Categorical data are given as frequencies and percentages. Outliers were identified via evaluation of the standardized residues and included whenever measurement errors could be excluded. Statistical comparisons between the two groups were performed using the Student's *t*-test. Associations with disease severity parameters were analyzed by using Pearson's correlation tests and corrected for age- and sex-related differences. Logistic regression was performed to investigate the relationship between clinical and laboratory markers [albuminuria, lyso-Gb3, NTproBNP, hsTNT, and interventricular septal wall diameter (IVSd)] and VEGFα and angiostatin serum levels, respectively. *p-value ≤0.05 was considered statistically significant.

## Results

### Protein expression of VEGFα and angiostatin in aortic rings of α-GAL-Tg/KO mice

**Figure 1:**
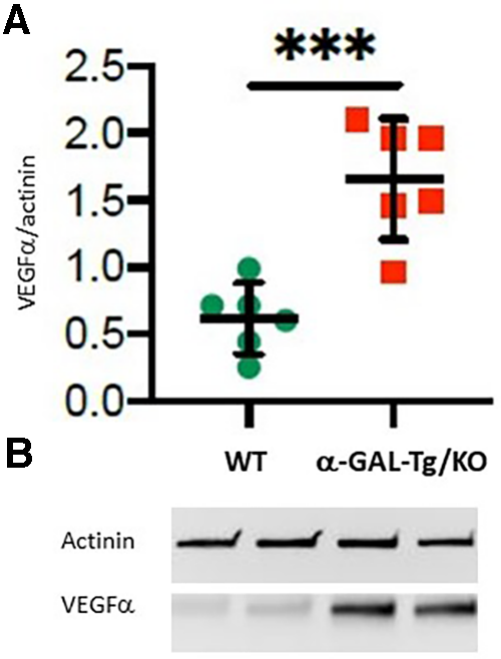
VEGFα protein expression in the aortic rings of α-GAL-Tg/KO mice. (**A**) Protein expression by Western blotting of VEGFα in the aortic rings of α-GAL-Tg/KO (*n* = 6) and WT mice (*n* = 6). The expression level of VEGFα was normalized to the internal control actinin and represented as the expression ratio. *p-value <0.05 was considered statistically significant, ***p < 0.001. (**B**) Characteristic Western blots for VEGFα expression compared with actinin as control.

Western blots with aortic lysates from six α-GAL-Tg/KO and six WT mice revealed a significant increase in VEGFα protein levels in α-GAL-Tg/KO mice compared with that in WT mice (signal intensity = 1.66 ± 0.35 vs. 0.62 ± 0.16); *p = 0.0009), as shown in Figure 1.

**Figure 2:**
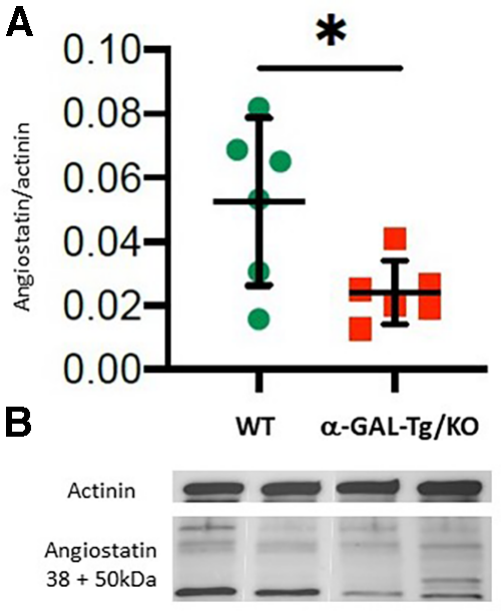
Angiostatin protein expression in the aortic rings of α-GAL-Tg/KO mice. (**A**) Protein expression by Western blotting of angiostatin in the aortic rings of α-GAL-Tg/KO (*n* = 6) and WT mice (*n* = 6). The expression level of angiostatin was normalized to the internal control actinin and represented as the expression ratio. *p-value <0.05 was considered statistically significant. (**B**) Characteristic Western blots for angiostatin expression compared with actinin as control.

Angiostatin expression showed a decrease in aortic tissue from α-GAL-Tg/KO mice compared with that from WT mice (signal intensity = 0.024 ± 0.007 vs. 0.053 ± 0.02, *p = 0.038), as shown in Figure 2.

### Immunohistochemical staining of VEGFα in aortic rings of α-GAL-Tg/KO mice

**Figure 3:**
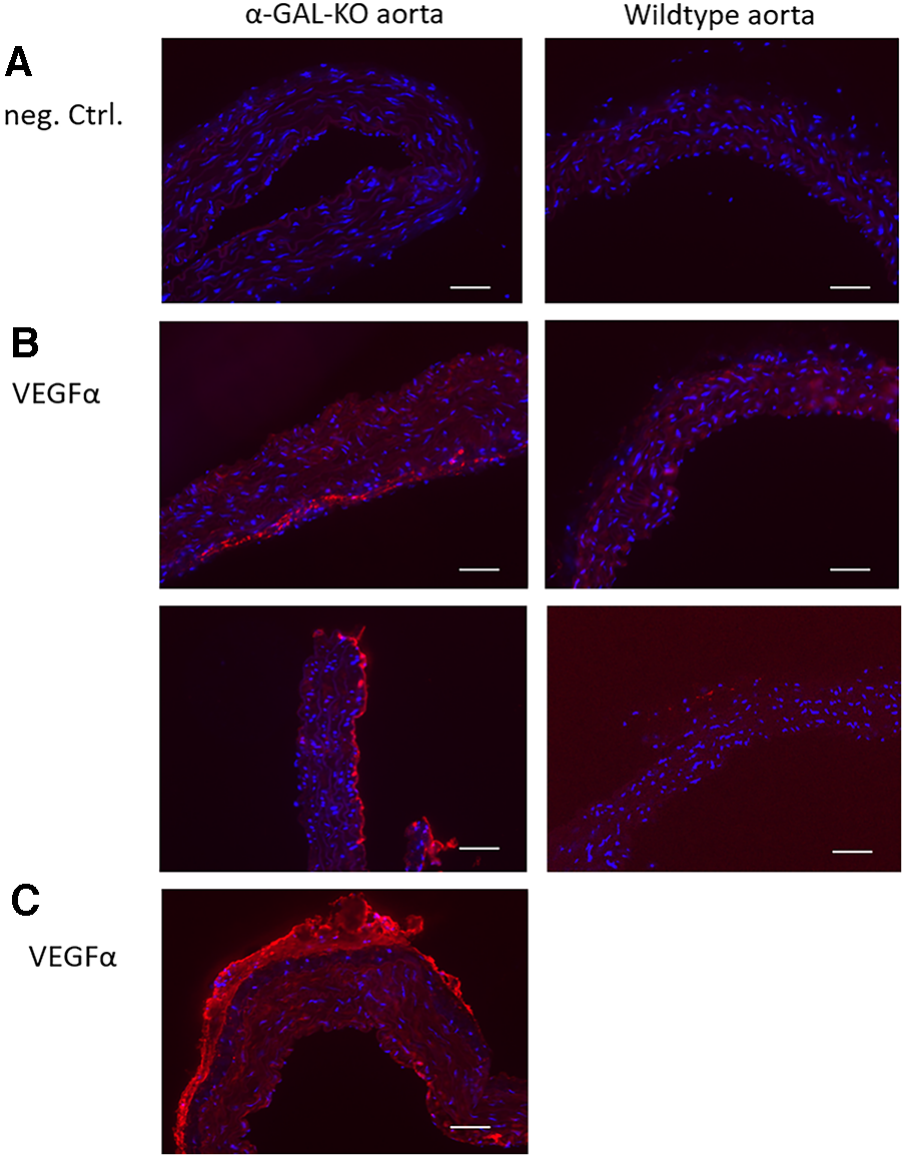
Immunohistochemical VEGFα staining of aortic rings from α-GAL-Tg/KO- and WT mice. A specific VEGFα signal could be detected in the adventitial layer of α-GAL-Tg/KO aortas. No VEGFα signal could be detected in the aortic rings from WT mice. (**A**) Negative control (staining without a primary antibody). (**B**) VEGFα signal in the aortic rings from α-GAL-Tg/KO mice (left) and WT mice (right). (**C**) An overview of a half VEGFα-stained aortic ring from a α-GAL-Tg/KO mouse. A similar VEGFα signal distribution pattern was detectable in all other aortic preparations from α-GAL-Tg/KO mice (not shown). Scale bars = 50 µm.

Immunohistochemical staining of aortic rings showed an adventitial VEGFα signal in α-GAL-Tg/KO mice. No VEGFα signal could be detected in WT mice aortas (Figure 3). In contrast to the results of the Western blot analysis, there were no detectable differences in angiostatin expression levels between α-GAL-Tg/KO and WT mice (not shown).

### VEGFα- and angiostatin serum levels in Fabry patients

#### Baseline characteristics

A total of 48 patients with Fabry’s disease with genetically confirmed mutations in the *GLA* gene [c.1277del (3×), c.500T>C, c.718del (4×), IVS1-6, p.717del (2×), p.A143T (3×), p.A230del (2×), p.A389V, p.C94S, p.E341K (2×), p.I384N (2×), p.I91T (2×), p.K213M (2×), p.L310V, p.L311P, p.L89del (2×), p.N139S, p.N215S (6×), p.205 T, p.Q327l (2×), p.R118C, p.R227Q, p.R227X (4×), p.R342, p.S247P] and 22 healthy controls were included in the study. Of these, 26 patients with Fabry’s disease presented with signs of cardiomyopathy, such as left ventricular hypertrophy combined with elevated serum markers of NTproBNP and/or hsTNT and/or positive LGE in CMR. In 22 patients, Fabry-associated kidney disease was detected. This was defined by biopsy and/or albuminuria without a potential other cause. Table 1 summarizes the baseline characteristics for male and female patients with Fabry’s disease and for the control group. As expected, patients with Fabry’s disease enrolled into this study suffered from a significantly increased rate of arrhythmias and dyspnea, resulting in a higher NYHA class, and presented with higher levels of cardiac and renal markers as the control group patients. Male patients with Fabry’s disease showed higher lyso-Gb3 levels than female patients, indicating a more severe phenotype in male patients, which was reflected by a higher percentage of male patients with cardiomyopathy and renal dysfunction. In accordance with this, the parameters of Fabry-associated cardiomyopathy and nephropathy were more prominent in male patients than in female patients.

**Table 1:** Baseline characteristics of patients with Fabry's disease and healthy controls.

*n* (%) or mean ± SD	Fabry’s disease	Fabry’s disease	Fabry’s disease	Control
	Total	Male	Female	M = 22/F = 2
*n*	48	24 (50%)	24 (50%)	22
Age (years)	41.3 ± 11.4*	39.6 ± 12.3	42.9 ± 10	30.8 ± 8.7
BMI (kg/m^2^)	24.4 ± 4.1	24.6 ± 3.9	24.2 ± 4.4	23.8 ± 1.8
Fabry nephropathy	22 (46%)**	15 (63%)	7 (29%)	0
Albuminuria <30 mg/L	26 (54%)	9 (38%)	17 (71%)	n.d.
Albuminuria 30–300 mg/L	13 (27%)	6 (25%)	7 (29%)	n.d.
Albuminuria >300 mg/L	9 (19%)	9 (38%)***	0	n.d.
Fabry cardiomyopathy	26 (54%)*	17 (71%)***	9 (38%)	0
Clinical presentation				
NYHA I	31 (65%)*	13 (54%)***	18 (75%)	22 (100%)
NYHA II	13 (27%)*	7 (29%)	6 (25%)	0
NYHA III	4 (8%)*	4 (17%)***	0	0
NYHA IV	0	0	0	0
Syncope	6 (13%)*	4 (17%)***	2 (8%)	0
ECG				
QTc (ms)	415 ± 24.3**	409.8 ± 27.3	420.7 ± 21.5	388 ± 15.5
Atrial fibrillation	5 (10%)*	4 (17%)***	1 (4%)	0
nsVT	5 (10%)*	3 (13%)***	2 (8%)	0
Laboratory values				
Lyso-Gb3 (ng/ml)	20.7 ± 20.9	35.9 ± 27.1****	5.4 ± 3.4	n.d.
NTproBNP (ng/L)	956.5 ± 1,427.2*	1,549 ± 2,489.1	364 ± 425.3	34.7 ± 16.8
hsTNT (pg/ml)	21.9 ± 21.7*	25.4 ± 22.1	18.3 ± 20.7	4.6 + 2
GFR (CKD) (ml/min)	78.7 ± 28.3	77.7 ± 35.5	79,7 ± 18	95.9 + 21
Creatinine (mg/dl)	1.2 ± 0.6	1.6 ± 0.9***	0.9 ± 0.2	1 + 0.1
Albuminuria (mg/L)^b^	557.2 ± 596.9	860.5 ± 729***	64 ± 33.5	n.d.
Cardiac imaging				
IVSd (mm)	13.1 ± 3.6**	14.7 ± 3.4***	11.4 ± 3.0	10.1 ± 0.7
PWd (mm)	12 ± 2.8**	13.5 ± 2.6***	10.4 ± 2.4	8.9 ± 1.1
LVEF >60%	40 (83%)	19 (79%)	21 (88%)	22 (100%)
LVEF 46%–60%	6 (13%)	4 (17%)	2 (8%)	0
LVEF <45%	1 (2%)	1 (4%)	0	0
LA (mm)	38.3 ± 6.5*	41.9 ± 6.5***	34.8 ± 6.2	34.4 ± 4.6
E/A	1.6 ± 0.4	1.5 ± 0.4	1.6 ± 0.5	1.8 ± 0.3
E/e’	10.7 ± 4.5*	12.9 ± 5.2***	8.6 ± 2.8	7.1 ± 1.2
LGE in CMR^a^	20 (50%)**	13 (65%)	7 (35%)	0
Medication				
ERT	29 (60%)	19 (79%)****	10 (21%)	0
Migalastat	1 (2%)	1 (4%)	0	0
ACE inhibitors/AT-1 blocker	18 (38%)	13 (54%)***	5 (21%)	0
Beta blockers	8 (17%)	5 (21%)	3 (13%)	0
Statins	9 (19%)	7 (29%)***	2 (8%)	0
Diuretics	8 (17%)	5 (21%)	3 (13%)	0
Concomitant diseases				
CAD	5 (10%)*	5 (21%)***	0	0
Diabetes	1 (2%)	1 (4%)	0	0
Hypertension	17 (35%)**	11 (46%)***	6 (25%)	0
Stroke	5 (10%)*	3 (13%)	2 (8%)	0

A, peak late transmitral filling velocity; ACE inhibitors, angiotensin-converting enzyme inhibitors; AT-1R, angiotensin I-receptor; BMI, body mass index; E, peak early transmitral filling velocity; e, early mitral annulus velocity; eGFR, estimated glomerular filtration rate; F, female; LA, left atrial; LVEF, left ventricular ejection fraction; M, male; *n*, total number; nsVT, non-sustained ventricular tachycardia; PWd, posterior wall diameter.

Values for continuous data are given as mean ± SD. Values for categorical data are given as counts and percentage of the total column number.

^a^ LGE in *n* = 40 receiving CMR.

^b^ Albuminuria in *n* = 22 with Fabry-associated nephropathy.

* *p*-value ≤0.05 vs. control.

** *p*-value ≤0.001 vs. control.

*** *p*-value ≤0.05 male vs. female patients with Fabry’s disease.

**** *p*-value ≤0.001 male vs. female patients with Fabry’s disease.

#### VEGFα and angiostatin serum levels in patients with Fabry’s disease compared with those in healthy controls

**Figure 4:**
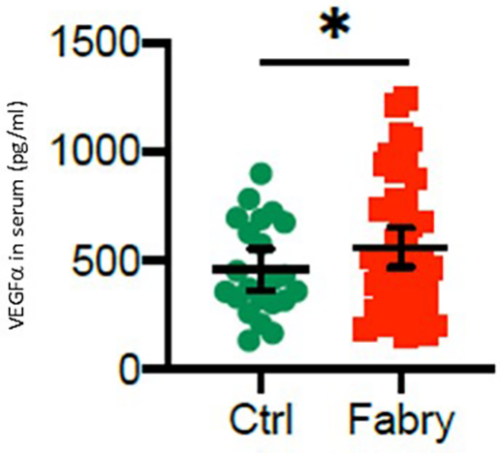
VEGFα serum level in patients with Fabry’s disease. Serum concentrations of VEGFα in 48 patients with Fabry’s disease and 22 healthy controls determined by using an enzyme-linked immunosorbent assay. *p-value <0.05 was considered statistically significant.

The serum levels of VEGFα were significantly upregulated in all patients with Fabry’s disease compared with that in healthy controls (708.5 ± 426.3 vs. 458.5 ± 181.5 pg/ml, *p = 0.048), as shown in Figure 4. This effect was more pronounced in male patients with Fabry’s disease (721.1 ± 420.5 pg/ml) than in female patients (695.9 ± 153.5 pg/ml), although a gender-specific analysis revealed no statistical significance.

**Figure 5:**
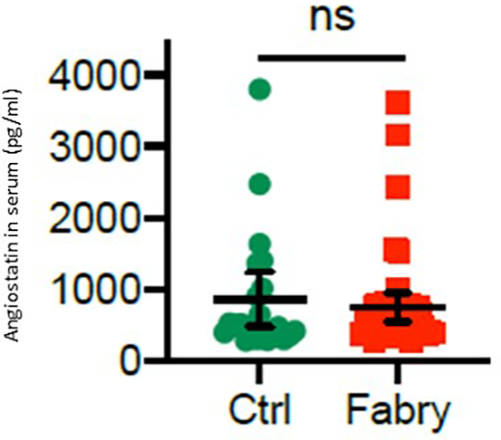
Angiostatin serum level in patients with Fabry’s disease. Serum concentrations of angiostatin in 48 patients with Fabry’s disease and 22 healthy controls determined by using an enzyme-linked immunosorbent assay.

An analysis of angiostatin serum levels revealed no statistical significance (747.6 ± 390.3 vs. 858.8 ± 599.3 pg/ml) either in male or in female patients with Fabry’s disease compared with those in controls (Figure 5). However, in female patients, there was a trend toward reduced angiostatin concentrations (581.6 ± 153.5 pg/ml), whereas this trend was not observed in male patients (913.6 ± 623.1 pg/ml).

#### Association of VEGFα and angiostatin serum levels with markers of disease severity in patients with Fabry’s disease

VEGFα levels were positively associated with increased albuminuria and elevated serum concentrations of NTproBNP and hsTNT in male patients with Fabry’s disease, whereas this effect could not be detected in female patients. Lyso-Gb3 and IVSd were not associated with higher VEGFα concentrations in these patients. No relationship could be detected between angiostatin serum concentrations and disease severity markers (Table 2).

**Table 2:** Association of different biomarkers for organ manifestation with VEGFα and angiostatin serum levels.

	Fabry’s disease Total (*n* = 48)		Fabry’s disease Male (*n* = 24)		Fabry’s disease Female (*n* = 24)	
	*r*	*p*-value	*r*	*p*-value	*r*	*p*-value
VEGFα (pg/ml) correlated to						
Lyso-Gb3 (ng/ml)	0.11	0.47	0.23	0.28	−0.19	0.37
NTproBNP (ng/l)	0.51	≤0.001	0.87	≤0.0001	0.2	0.34
hsTNT (pg/mL)	0.05	0.75	0.41	0.048*	−0.13	0.54
Albuminuria (mg/L)	0.47	≤0.001	0.82	≤0.0001	−0.16	0.46
IVSd (mm)	−0.03	0.84	0.1	0.63	−0.15	0.48
Angiostatin (pg/ml) correlated to						
Lyso-Gb3 (ng/ml)	0.28	0.051	0.19	0.38	0.2	0.34
NTproBNP (ng/L)	−0.002	0.99	−0.04	0.85	0.1	0.64
hsTNT (pg/ml)	−0.03	0.84	−0.15	0.48	0.12	0.59
Albuminuria (mg/L)	0.15	0.3	0.09	0.68	−0.1	0.64
IVSd (mm)	0.10	0.48	0.01	0.96	0.05	0.83

*r*, Pearson's correlation coefficient.

Results from the correlation tests of laboratory values and markers of organ manifestation in 48 patients with Fabry’s disease. All values were corrected for age- and sex-related differences.

* *p*-value ≤0.05 was considered statistically significant.

No statistically significant influence of concomitant medication on VEGF and angiostatin serum levels could be detected in patients with Fabry’s disease. Also for enzyme replacement therapy (ERT), there was no significant effect on VEGF and angiostatin concentrations, although there was a trend toward lower VEGF concentrations in patients with Fabry’s disease under ERT compared with those in therapy-naive patients with Fabry’s disease (data not provided).

## Discussion

### Main findings

In this study, we found increased endothelial VEGFα and reduced angiostatin levels in the aortic rings of mice with genetically defective α-GAL activity, as well as increased serum levels of VEGFα in patients with Fabry’s disease, highlighting the role of endothelial cell activation and oxidative stress in Fabry's disease.

### Overexpression of VEGFα

Our data demonstrate for the first time a significant increase in VEGFα protein expression in aortic lysates from α-GAL- Tg/KO mice compared with those from WT mice. In Fabry's disease, storage of Gb3, as a result of deficient α-GAL A activity, was identified to induce oxidative stress in endothelial cells (21, 22), leading to an activation of inflammatory and pro-angiogenic reactions (23) *in vitro* and *in vivo*, including changes in the pattern of circulating miRNA (24). In this context, we analyzed the expression of the most potent pro-angiogenic factor VEGFα and its counter player angiostatin in the aortic rings of the Fabry R301Q transgenic/KO mouse model and compared this with the expression in the serum of patients with Fabry’s disease and healthy controls. In line with our data, treatment of bovine aortic endothelial cells with Gb3 induced the expression of angiogenic factors such as TGfβ, VEGFR2, VEGFα, and FGF2 (3, 25), demonstrating a pivotal role of angiogenetic factors in Gb3-induced vasculopathy. Further studies using the α-GAL-Tg/KO mouse model revealed a renal overexpression of VEGF and TGFβ in the presence of Fabry nephropathy (3).

In addition, immunohistochemical staining for VEGFα was performed in the aortic rings of α-GAL-Tg/KO and WT mice, which showed a higher degree of staining of VEGFα in α-GAL-Tg/KO mice. Although smooth muscle cells have been shown to be the major source of VEGFα expression and secretion (26), and accordingly in a model of hypertensive rats, VEGFα expression was mainly distributed in the outer to middle layers of the media (27), our data indicate that the VEGFα signal in mice aortic tissue was mainly increased in the adventitial layer. Whether this is a species-specific effect is not known. However, our observations are supported by a study in human atherosclerotic arteries analyzing the localization of different VEGF isoforms, also showing the most prominent staining for VEGFα in the adventitia along with some staining of the media (28).

To reveal the relevance of VEGFα expression seen in this *in vitro* model, we also measured VEGFα concentrations in the serum of 48 patients with Fabry’s disease and 22 healthy controls, which showed significantly higher VEGFα concentrations in the Fabry’s disease cohort. In line with other studies demonstrating VEGFα overexpression in patients with Fabry’s disease with cutaneous and systemic manifestations, this effect was more pronounced in male patients (29). This may be explained by the fact that male patients presented with a more severe clinical phenotype and a higher lyso-Gb3 level, which is usually attributed to lower enzyme activities in male patients compared with female patients. In this context, a proteomics-based analysis recently showed a correlation of VEGFα with lyso-Gb3 and residual enzyme activity in classical Fabry’s disease (30). Although an association with a higher lyso-Gb3 level could not be confirmed in our cohort, we show that VEGFα was positively associated with albuminuria, NTproBNP, and hsTNT, as markers for organ manifestation in Fabry's disease. However, this relationship was solely seen in male patients with Fabry’s disease.

No statistically relevant influence of co-medication on VEGFα levels could be found, although there was a trend toward lower VEGFα serum concentrations in patients with Fabry’s disease under ERT compared with those in the small group of untreated patients with Fabry’s disease. This may indicate a potentially beneficial effect of ERT, which has to be further evaluated in a larger cohort of patients with Fabry’s disease.

### Reduced expression of angiostatin

Angiostatin is an internal fragment of plasminogen and is generated by proteolytic cleavage through matrix metalloproteinases (MMP) and urokinase plasminogen activator (uPA) (31, 32). It acts as a potent inhibitor of angiogenesis and attenuates VEGF expression, resulting in the inhibition of extracellular matrix formation and migration (10, 11). Recently, we showed altered MMP9 and angiostatin levels in the serum of patients with Fabry’s disease compared with healthy controls, suggesting a higher extracellular matrix turnover in Fabry's disease (16). Accordingly, we found a suppressed angiostatin expression in aortic rings of the α-GAL-Tg/KO mouse model. In the serum of patients with Fabry’s disease, we also observed a trend toward reduced angiostatin serum levels; however, this did not reach statistical significance.

## Conclusions

An overexpression of VEGFα in aortic tissue of the Fabry mouse model and a corresponding higher serum level of VEGFα, especially in male patients with a progressive state of Fabry's disease, support the hypothesis of an underlying vasculopathy. There is growing evidence that vascular damage induced by lipid storage or substrate-independent mechanisms is an underlying pathophysiologic feature in the development of progressive organ manifestation. In this context, our findings call for further research determining whether oxidative stress and endothelial cell activation are suitable therapeutic targets in Fabry's disease.

## Strengths and limitations

One of the strengths of the study lies in the translational approach quantifying VEGFα in the vascular rings of an established murine model of Fabry's disease and in the serum of genotyped patients with Fabry’s disease. However, a limitation is that data were obtained only from a single center, which necessitates independent validation. The other limitations are as follows: patients with Fabry’s disease were approximately 10 years older than the control group patients and only two female patients were included in the control group. Therefore, age- and sex-related influences on VEGFα and angiostatin levels could not be sufficiently validated. Also, residual enzyme activity α-GAL was not explicitly measured in our cohort.

## Data Availability

The raw data supporting the conclusions of this article will be made available by the authors, without undue reservation.
